# Anti-Obesity and Anti-Diabetic Effects of Acacia Polyphenol in Obese Diabetic KKAy Mice Fed High-Fat Diet

**DOI:** 10.1093/ecam/nep241

**Published:** 2011-04-14

**Authors:** Nobutomo Ikarashi, Takahiro Toda, Takehiro Okaniwa, Kiyomi Ito, Wataru Ochiai, Kiyoshi Sugiyama

**Affiliations:** Department of Clinical Pharmacokinetics, Hoshi University, Tokyo, Japan

## Abstract

Acacia polyphenol (AP) extracted from the bark of the black wattle tree (*Acacia meansii*) is rich in unique catechin-like flavan-3-ols, such as robinetinidol and fisetinidol. The present study investigated the anti-obesity/anti-diabetic effects of AP using obese diabetic KKAy mice. KKAy mice received either normal diet, high-fat diet or high-fat diet with additional AP for 7 weeks. After the end of administration, body weight, plasma glucose and insulin were measured. Furthermore, mRNA and protein expression of obesity/diabetic suppression-related genes were measured in skeletal muscle, liver and white adipose tissue. As a result, compared to the high-fat diet group, increases in body weight, plasma glucose and insulin were significantly suppressed for AP groups. Furthermore, compared to the high-fat diet group, mRNA expression of energy expenditure-related genes (PPAR**α**, PPAR**δ**, CPT1, ACO and UCP3) was significantly higher for AP groups in skeletal muscle. Protein expressions of CPT1, ACO and UCP3 for AP groups were also significantly higher when compared to the high-fat diet group. Moreover, AP lowered the expression of fat acid synthesis-related genes (SREBP-1c, ACC and FAS) in the liver. AP also increased mRNA expression of adiponectin and decreased expression of TNF-**α** in white adipose tissue. In conclusion, the anti-obesity actions of AP are considered attributable to increased expression of energy expenditure-related genes in skeletal muscle, and decreased fatty acid synthesis and fat intake in the liver. These results suggest that AP is expected to be a useful plant extract for alleviating metabolic syndrome.

## 1. Introduction

Acacia is an evergreen tree belonging to the genus *Acacia* in the family legume, and is widely found in the Australian and African continents. The extract of *Acacia catechu* duramen is called gambir, and has long been used as an astringent and anti-bacterial to treat stomatitis and diarrhea in Japan and China. Studies have also reported that the powdered seeds of *Acacia catechu* and *Acacia milanoxylon* exhibit hypoglycemic actions by increasing insulin secretion in non-diabetic rats and rabbits [1, 2]. Furthermore, in Europe, acacia polyphenol (AP) extracted from the bark of the black wattle tree (*Acacia meansii*) has been used to eliminate wine sediment. Australian aborigines also eat the young leaves and beans of *Acacia mollissima*.

AP is rich in unique catechin-like flavan-3-ols, such as robinetinidol and fisetinidol [3–6]. In recent years, polyphenols have been reported to possess various pharmacological actions, including anti-obesity actions [7–9], anti-diabetic actions [10–12], anti-cancer actions [13, 14] and anti-allergic actions [15]. While polyphenol-rich AP may possess various pharmacological actions like other polyphenols, no studies have yet clarified the pharmacological actions of AP.

In the present study, to investigate the anti-obesity and anti-diabetic actions of AP, a high-fat diet was administered to KKAy mice, an obese type-II diabetes model, to induce severe obesity and diabetes. The effects of AP on obesity and diabetes were then studied. To elucidate the mechanisms underlying the suppression of obesity and diabetes, the effects of AP on the expression of genes related to obesity and diabetes suppression in skeletal muscle, liver and white adipose tissue were investigated.

## 2. Materials and Methods

### 2.1. Hot Water Extraction from Acacia Bark

AP was donated by Mimozax Co., Ltd. (Hiroshima, Japan), and was prepared according to the methods reported by Cutting [16]. Briefly, the powdered bark of South African *A. mollissima* was pulverized and extracted for 30 min in a 10-fold volume of hot water (100°C) and then dried using a spray drier. The polyphenol content of the present product as measured by the Stiasny reaction was 79.0% [17–19]. Average molecular weight of AP is 1250 (300–3000) [20, 21], and robinetinidol and fisetinidol are the major ingredients [3–6].

### 2.2. Materials

Powdered regular low-fat diet (LF) (10% lard, D12450B) and powdered high-fat diet (HF) (60% lard, D12492) were purchased from Research Diets (New Brunswick, NJ, USA). Primers were purchased from Invitrogen (Tokyo, Japan). The ECL Plus Western Blotting Detection System was purchased from GE Healthcare (Chalfont St Giles, UK). Anti-rabbit ACO antibody and anti-rabbit UCP3 antibody were purchased from Abcam (Cambridge, UK). Anti-rabbit CPT1 antibody was purchased from Santa Cruz Biotechnology (Santa Cruz, CA, USA). Anti-rabbit *β*-actin antibody was purchased from BioLegend (San Diego, CA, USA). Anti-rabbit IgG (whole molecule) peroxidase conjugate was purchased from Sigma-Aldrich (St Louis, MO, USA). All other reagents were of the highest commercially available grade.

### 2.3. Animals, Diets and Treatment

Male KKAy mice (5-weeks old) were purchased from Clea Japan (Tokyo, Japan). Each mouse was caged separately and kept at room temperature (22 ± 1°C) and 55 ± 10% humidity with 12 h of light (artificial illumination: 08 : 00–20 : 00). Mice that had been acclimatized for 1 week were divided into four groups. Each group was provided with ad libitum access to either LF, HF, HF containing 2.5% (w/w) AP (HF/AP 2.5%) or HF containing 5.0% (w/w) AP (HF/AP 5.0%) for 7 weeks. Food intake was measured once weekly. Following this feeding period, after 17 h of fasting, a blood sample was collected using heparin from the abdominal vena cava under diethyl ether-induced anesthesia. The liver, white adipose tissue (around the testes, retroperitoneum and kidney) and skeletal muscle were also removed and placed in either neutral-buffered formalin or liquid nitrogen. The present study was conducted in accordance with the Guiding Principles for the Care and Use of Laboratory Animal, as adopted by the Committee on Animal Research at Hoshi University.

### 2.4. Blood Analysis

Blood samples were centrifuged (1000 g for 15 min at 4°C), and plasma stored at −80°C until assay. Plasma glucose concentrations were enzymatically quantified using a Glucose CII Test (Wako Pure Chemical Industries, Osaka, Japan). Plasma insulin concentrations were measured according to the protocol described by the manufacturer of the Mouse Insulin ELISA Kit (Shibayagi, Gunma, Japan). Plasma glutamate oxalacetate transaminase (GOT) and glutamate pyruvate transaminase (GPT) concentrations were quantified using Transaminase CII Test (Wako Pure Chemical Industries).

### 2.5. Homeostasis Model Assessment of Insulin Resistance Index

Homeostasis model assessment of insulin resistance (HOMA-IR), an index of insulin resistance, was calculated according to the following equation using fasting insulin and glucose concentrations [22]:

(1) $$\begin{array}{cc} \text{HOMA-IR} & =\text{fasting}\;\text{insulin}\;\left(\mu \text{U}\;{\text{ml}}^{-1}\right)\\ & \;\times \text{fasting}\;\text{glucose}\;\left(\text{mmol}\;{\text{l}}^{-1}\right)/22.5. \end{array}$$

### 2.6. Measurement of Liver Triglyceride/Cholesterol Content

Liver triglyceride and cholesterol content were measured as described previously [23, 24]. Briefly, a portion (100 mg) of liver tissue was homogenized in phosphate buffer saline (pH 7.4, 1 ml). The homogenate (0.2 ml) was extracted with isopropyl alcohol (1 ml), and the extract was analyzed using a Triglyceride *E*-Test (Wako Pure Chemical Industries) to determine liver triglyceride content. The homogenate (0.2 ml) was extracted with chloroform-methanol (2 : 1, 1 ml), and the extract was concentrated under a nitrogen stream. The residue was dissolved in isopropyl alcohol and analyzed using a Cholesterol *E*-Test (Wako Pure Chemical Industries).

### 2.7. Preparation of Slide for Histopathology

#### 2.7.1. Hematoxylin—Eosin Staining

Liver and epididymal fat were fixed in 10% neutral-buffered formalin. After trimming the tissues, liver and epididymal fat were embedded in paraffin using a tissue processor. Sections were taken in 3-4 *μ*m thicknesses, and stained with HE solution for microscopy. Average surface area for epididymal white adipocytes was measured using Image J software (National Institute of Mental Health, Bethesda, MD, USA) [25, 26].

#### 2.7.2. Oil red O Staining

After frozen liver sections about 7 *μ*m thick were prepared using a cryostat apparatus, oil red O staining was conducted for microscopy.

### 2.8. RNA Preparation from Tissue Samples

RNA was extracted from about 50 mg of frozen skeletal muscle using TRI reagent. RNA was extracted from about 15 mg of frozen liver using the RNeasy Mini Kit. RNA was extracted from about 75 mg of frozen epididymal white adipose tissue using the RNeasy Lipid Tissue Mini Kit. RNA extraction was performed according to the protocol for the TRI Reagent, RNeasy Mini Kit and RNeasy Lipid Tissue Mini Kit. The resulting solution was diluted 50-fold using TE buffer, and purity was confirmed and RNA concentration (*μ*g ml^−1^) was calculated by measuring absorbance at 260 and 280 nm using a U-2800 spectrophotometer (Hitachi High Technologies, Tokyo, Japan).

### 2.9. Real-time RT-PCR

A high-capacity cDNA synthesis kit was used to synthesize cDNA from 1 *μ*g of RNA. TE buffer was used to dilute the cDNA 20-fold to prepare cDNA TE buffer solution. The expression of target genes was detected by preparing primers listed in Table 1 and performing real-time RT-PCR. To each well of a Multiplate PCR Plates 96-well clear (Bio-Rad Laboratories), 25 *μ*l of iQ SYBR Green Supermix, 3 *μ*l of forward primer of target gene (5 pmol *μ*l^−1^), 3 *μ*l of reverse primer (5 pmol *μ*l^−1^), 4 *μ*l of cDNA TE buffer solution and 15 *μ*l of RNase-free water were added. With regard to *β*-actin, a housekeeping gene, 4 *μ*l of a cDNA TE buffer solution that was prepared by diluting above-mentioned solution 20-fold using TE buffer was used. Denaturation temperature was set at 95°C for 15 s, annealing temperature at 56°C for 30 s and elongation temperature at 72°C for 30 s. Fluorescence intensity of the amplification process was monitored using the My iQ^TM^ Single Color Real-time RT-PCR Detection System (Bio-Rad Laboratories). mRNA expressions were normalized using *β*-actin. All mRNA expressions were expressed in relation to the average expression of the LF group (100%).

### 2.10. Western Blotting Analysis

Leg muscle (100 mg) was homogenized using dissecting buffer (0.3 M sucrose, 25 mM imidazole, 1 mM EDTA, pH 7.2, containing 8.5 *μ*M leupeptin, and 1 mM phenylmethylsulfonyl fluoride), and the homogenates were centrifuged at 500 g for 10 min at 4°C [27, 28]. Total protein content in the supernatants was determined by the Lowry method [29] using bovine serum albumin as a standard.

Electrophoresis was performed using the method described by Laemmli [30]. Using the loading buffer (84 mM Tris, 20% glycerol, 0.004% bromophenol blue, pH 6.3, 4.6% SDS and 10% 2-mercaptoethanol), protein was diluted 2-fold, boiled for 5 min and applied to 12.5% polyacrylamide gel (ATTO Corp., Tokyo, Japan). After electrophoresis, the isolated proteins were transferred to a PVDF membrane (ATTO Corp.) using CompactBLOT (AE-7500, ATTO Corp.). After blocking for 1 h using 1.0% skim milk, the resulting membrane was reacted for 1 h at room temperature with anti-rabbit CPT1 antibody (1 : 100), ACO antibody (1 : 200), UCP3 antibody (1 : 1,000) or *β*-actin antibody (1 : 200). After washing the membrane using TBS-TWEEN (Tris-HCl 20 mM, NaCl 137 mM and Tween 20 0.1%, pH 7.6), the resulting membrane was reacted for 1 h at room temperature with anti-rabbit IgG peroxidase conjugate. After washing, the membrane was reacted with the ECLplus detection reagent and visualized with an LAS-3000 mini luminoimage analyzer (Fuji Film, Tokyo, Japan). Protein levels were normalized against *β*-actin. All protein expressions are given as percentages compared to the LF group (100%).

### 2.11. Statistical Analysis

Numerical data are expressed as means ± standard deviation. The significance of differences was examined using ANOVA, followed by Tukey's test. Values of *P* < .05 were considered significant.

## 3. Results

### 3.1. Body Weight and Visceral Fat Accumulation

During the 7-week experimental period, food intake was measured once weekly. Mean energy intake was significantly higher for the HF group than for the LF group. No significant difference existed in energy intake between the HF, HF/AP 2.5% and HF/AP 5.0% groups (Table 2).

At the end of administration, body weight was significantly higher for the HF group than for the LF group. AP administration significantly suppressed HF-induced body weight increases. In particular, the degree of body weight increase was comparable between the HF/AP 5.0% and LF groups (Table 2).

Furthermore, visceral white adipose tissue weight was significantly higher for the HF group than for the LF group. AP significantly suppressed this increase in a dose-dependent manner (Table 2).

**Figure 1:**
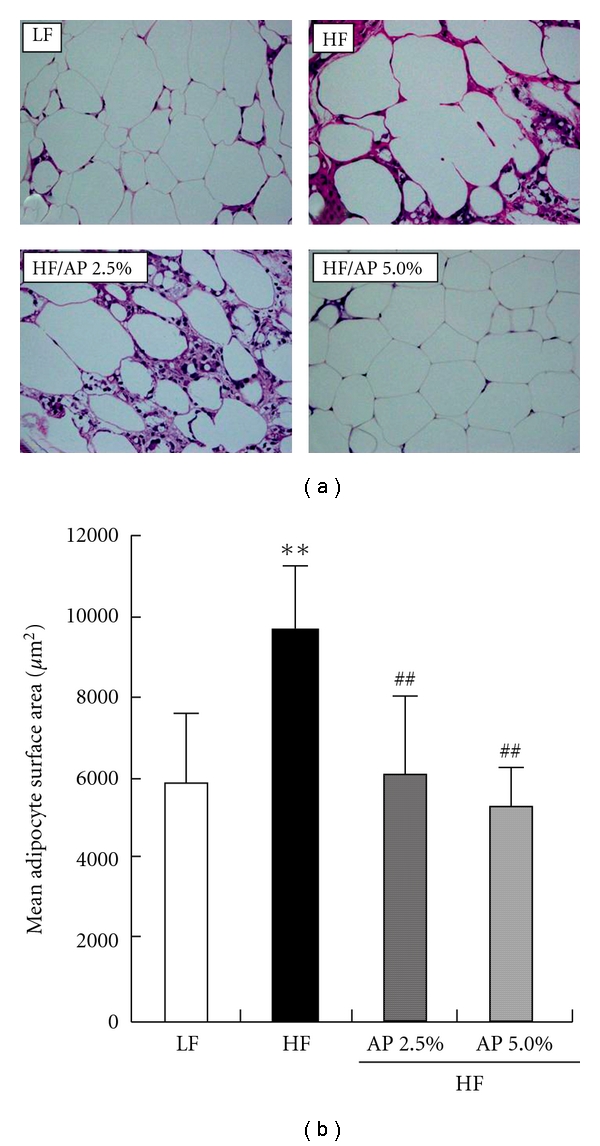
Histology of epididymal adipose tissue (a) and mean adipocyte surface area (b). KKAy mice were given a LF, a HF, HF containing 2.5% AP (HF/AP 2.5%) or HF containing 5.0% AP (HF/AP 5.0%) for 7 weeks. White adipose tissue around the testis was removed, fixed in 10% neutral-buffered formalin, paraffinized and stained using HE (a). Mean surface area for epididymal white adipocytes was measured using Image J software (b). Details of the diets are given in the “Materials and Methods” section. Data represent means ± SDs for seven sections per group. Tukey's test: **P < .01 versus LF; ^##^ *P* < .01 versus HF.

HE staining of epididymal white adipose tissue showed that mature adipocytes for the HF group were hypertrophied, with numerous immature adipocytes in the stroma. Conversely, HF-induced hypertrophy of mature adipocytes was not seen for the HF/AP 5.0% group, and the size of adipocytes and number of immature adipocytes were comparable to those for the LF group (Figure 1).

### 3.2. Liver Lipid Accumulation

Table 2 shows liver weight and liver lipid content. At the end of administration, liver weight was about 1.8 times greater for the HF group than for the LF group. In addition, liver triglyceride and cholesterol levels were significantly higher for the HF group when compared to the LF group. For both HF/AP 2.5% and HF/AP 5.0% groups, increased liver weight and triglyceride and cholesterol accumulation were significantly suppressed.

**Figure 2:**
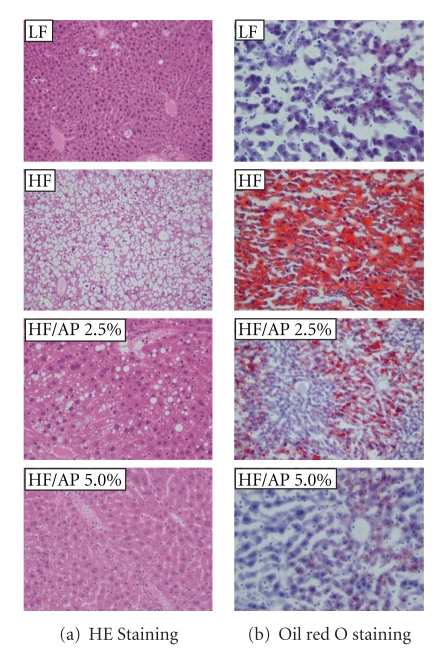
Representative histological sections of liver stained with HE (a) or oil red O (b). KKAy mice were given a LF, a HF, HF containing 2.5% AP (HF/AP 2.5%) or HF containing 5.0% AP (HF/AP 5.0%) for 7 weeks. The liver was removed, fixed in 10% neutral-buffered formalin, paraffinized and stained using HE (a). A cryostat was also used to prepare tissue sections for staining with oil red O (b). Details of the diets are given in the “Materials and Methods” section.

The liver was subjected to HE staining and oil red O staining, and the HF group displayed large fat droplets throughout liver lobules. However, HF/AP 2.5% and HF/AP 5.0% groups showed smaller fat droplets in individual hepatocyte and fewer fat droplets throughout liver lobules, thus indicating suppression of fatty liver (Figure 2).

### 3.3. Plasma Glucose, Insulin, GOT and GPT Concentration

Table 2 shows fasting plasma glucose, plasma insulin, GOT and GPT. Plasma glucose and plasma insulin levels were significantly higher for the HF group than for the LF group. Conversely, plasma glucose and insulin levels for the AP groups decreased to levels similar to those for the LF group, and HOMA-IR index in the HF/AP 2.5% and HF/AP 5.0% groups was significantly decreased compared to the HF group. Plasma GOT and GPT, indicators of hepatopathy, were significantly higher for the HF group than for the LF group. AP administration significantly suppressed increases in GOT and GPT.

### 3.4. mRNA and Protein Expression in the Skeletal Muscle

**Figure 3:**
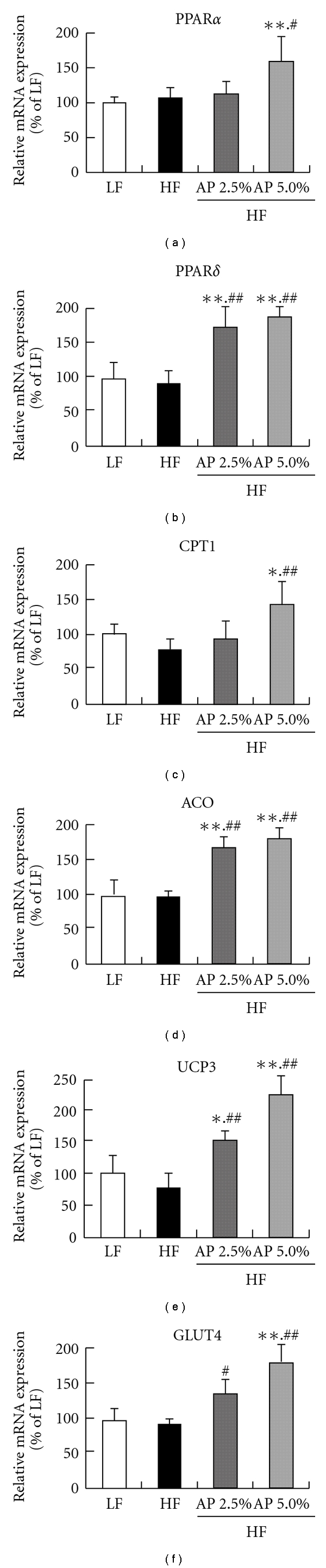
Effect of acacia polyphenol on mRNA expression in skeletal muscle. KKAy mice were given a LF, HF, HF containing 2.5% AP (HF/AP 2.5%) or HF containing 5.0% AP (HF/AP 5.0%) for 7 weeks. Skeletal muscle was removed, and mRNA expressions were measured using real-time RT-PCR, using *β*-actin as a housekeeping gene. All mRNA expressions are given as percentages compared to the LF group (100%). Details of the diets are given in the “Materials and Methods” section. Data represent means ± SDs for six mice per group. Tukey's test: *P < .05 versus LF; **P < .01 versus LF; ^#^ *P* < .05 versus HF; ^##^ *P* < .01 versus HF.

Real-time RT-PCR was performed to measure the mRNA expression of energy expenditure-related genes (peroxisome proliferator-activated receptor (PPAR)*α*, PPAR*δ*, carnitine palmitoyl-transferase 1 (CPT1), acyl CoA oxidase (ACO) and uncoupling protein 3 (UCP3)) and glucose transporter 4 (GLUT4) in skeletal muscle, an important organ for energy expenditure (Figure 3). No significant difference existed in all genes between LF and HF groups. The mRNA expression of PPAR*α* for the HF/AP 5.0% group was significantly higher (1.5 times) than that for the HF group. In addition, mRNA expression of PPAR*δ* for the HF/AP 2.5% and HF/AP 5.0% groups was 1.8 and 2.0 times higher when compared to the HF group. Moreover, AP significantly increased mRNA expression of CPT1, ACO and UCP3. In particular, compared to the HF group, mRNA expression of UCP3 for the HF/AP 2.5% and HF/AP 5.0% groups was about 2 and 3 times greater, respectively. AP administration increased mRNA expression of GLUT4 (abundantly expressed in skeletal muscle), and mRNA expression of GLUT4 for the HF/AP 5.0% group was about double that for the HF group.

**Figure 4:**
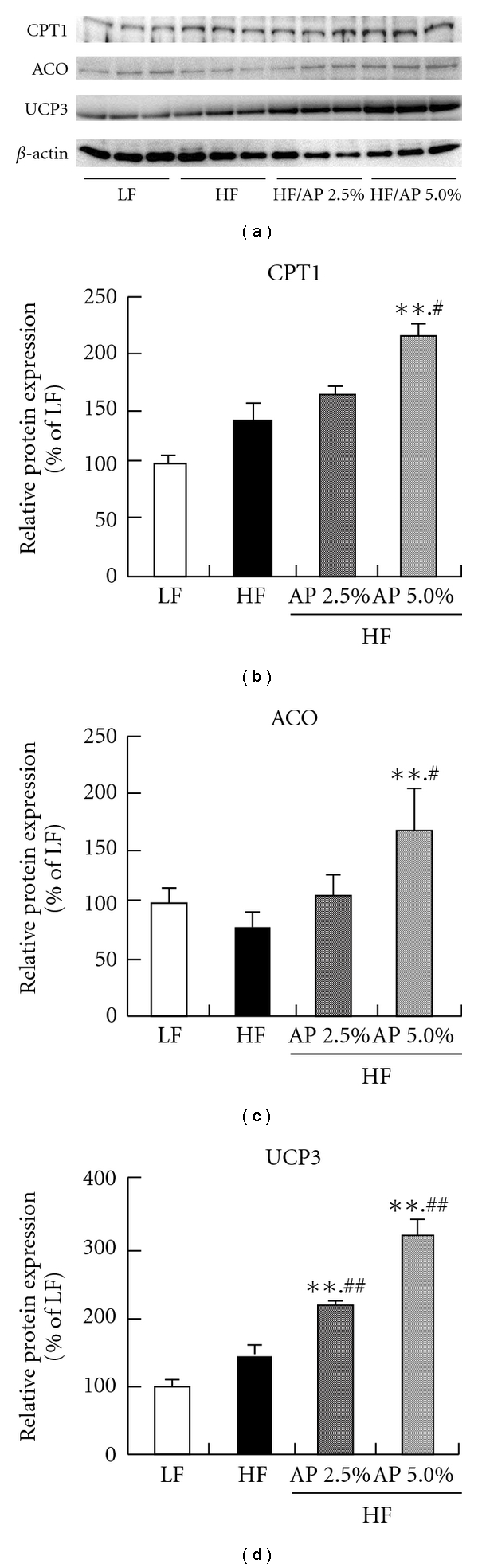
Effect of acacia polyphenol on protein expression of CPT1, ACO and UCP3 in skeletal muscle. KKAy mice were given a LF, a HF, HF containing 2.5% AP (HF/AP 2.5%) or HF containing 5.0% AP (HF/AP 5.0%) for 7 weeks. The skeletal muscle was removed, and protein expression was measured by western blotting, using *β*-actin as a housekeeping gene. All protein expressions are given as percentages compared to the LF group (100%). Details of the diets are given in the “Materials and Methods” section. Data represent means ± SDs for six mice per group. Tukey's test: **P < .01 versus LF; ^#^ *P* < .05 versus HF; ^##^ *P* < .01 versus HF.

Protein expressions of CPT1, ACO and UCP3 were quantified by western blotting, and AP administration significantly increased the expression of all proteins (Figure 4). These increases also correlated to increased mRNA expressions.

### 3.5. mRNA Expression in the Liver

**Figure 5:**
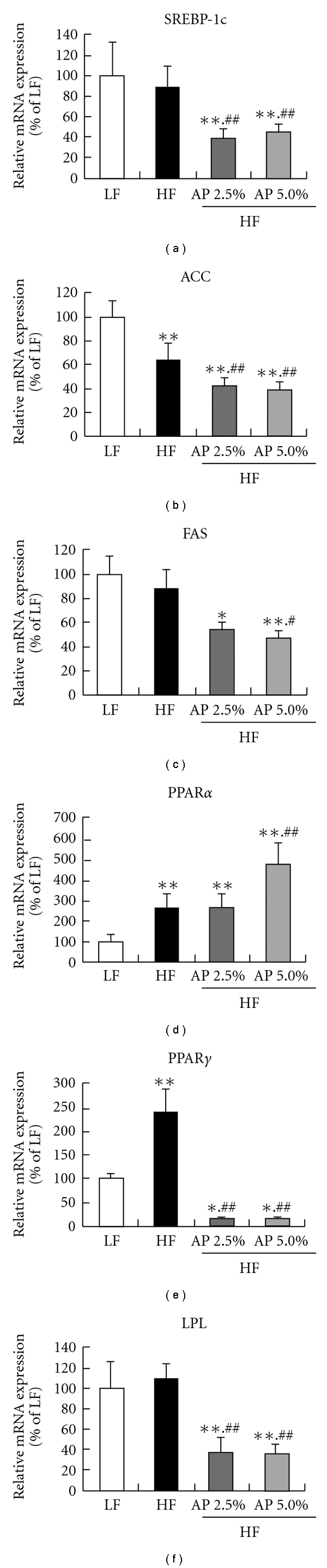
Effect of acacia polyphenol on mRNA expression in the liver. KKAy mice were given a LF, a HF, HF containing 2.5% AP (HF/AP 2.5%) or HF containing 5.0% AP (HF/AP 5.0%) for 7 weeks. The liver was removed and mRNA expression was measured by real-time RT-PCR using *β*-actin as a housekeeping gene. All mRNA expressions are given as percentages compared to the LF group (100%). Details of the diets are given in the “Materials and Methods” section. Data represent means ± SDs for six mice per group. Tukey's test: *P < .05 versus LF; **P < .01 versus LF; ^#^ *P* < .05 versus HF; ^##^ *P* < .01 versus HF.

In the liver, expressions of fatty acid synthesis-related genes (sterol regulatory element-binding protein (SREBP)-1c, acetyl-CoA carboxylase (ACC) and fatty acid synthase (FAS)), a fatty acid decomposition-related gene (PPAR*α*) and fat intake-related genes (PPAR*γ* and lipoprotein lipase (LPL)) were measured by real-time RT-PCR (Figure 5). The mRNA expressions of SREBP-1c, ACC and FAS were significantly lower for HF/AP 2.5% and HF/AP 5.0% groups than for the HF group. In addition, mRNA expression of PPAR*α* was significantly higher for the HF/AP 2.5% and HF/AP 5.0% groups than for the HF group. Furthermore, mRNA expressions of PPAR*γ* and LPL, which are related to liver fat accumulation, were significantly lower when compared to the HF group.

### 3.6. mRNA Expression in White Adipose Tissue

**Figure 6:**
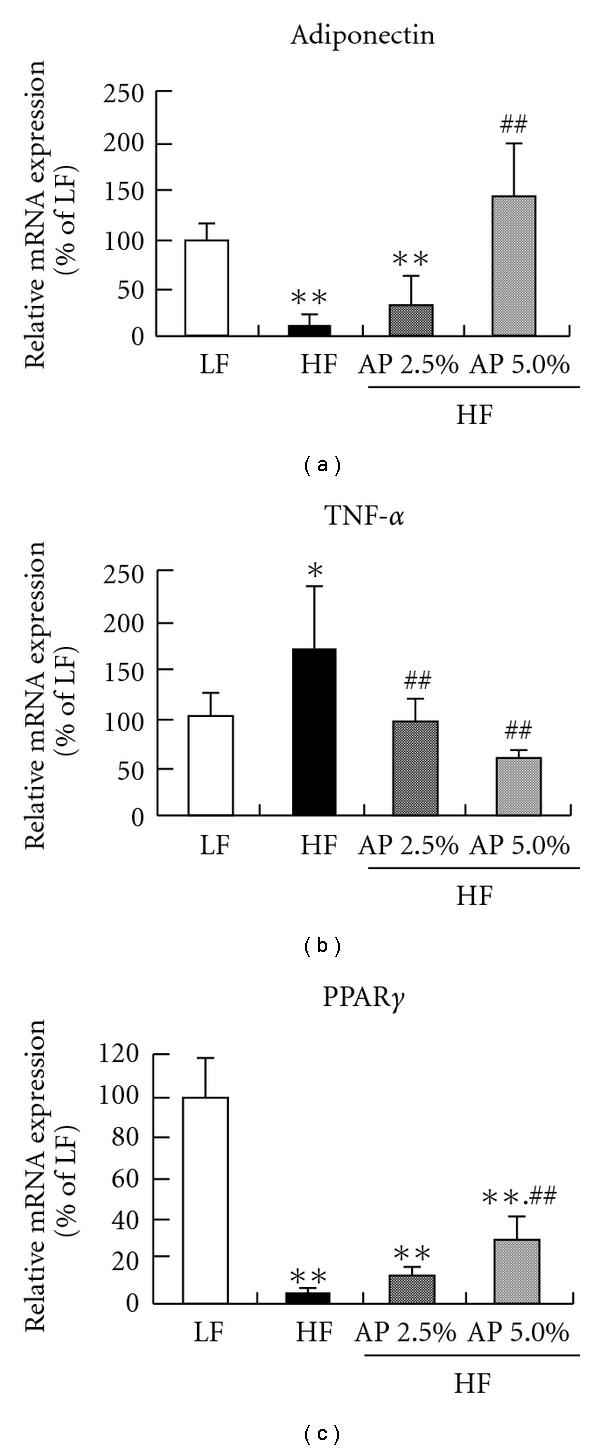
Effect of acacia polyphenol on mRNA expression in epididymal white adipose tissue. KKAy mice were given a LF, HF, HF containing 2.5% AP (HF/AP 2.5%) or HF containing 5.0% AP (HF/AP 5.0%) for 7 weeks. White adipose tissue around the testis was removed, and mRNA expression was measured by real-time RT-PCR, using *β*-actin as a housekeeping gene. All mRNA expressions are given as percentages compared to the LF group (100%). Details of diets are given in the “Materials and Methods” section. Data represent means ± SDs for six mice per group. Tukey's test: *P < .05 versus LF; **P < .01 versus LF; ^##^ *P* < .01 versus HF.

The mRNA expressions of adipocytokines (adiponectin and tumor necrosis factor (TNF)-*α*), which are produced in white adipose tissue and play important roles in obesity and diabetes, and PPAR*γ* were measured by real-time RT-PCR (Figure 6). The results showed that mRNA expression of adiponectin was significantly lower for the HF group than for the LF group, while mRNA expression of TNF-*α* was significantly higher. Conversely, mRNA expression of adiponectin was significantly higher for the HF/AP 2.5% and HF/AP 5.0% groups in a dose-dependent manner when compared to the HF group, but mRNA expression of TNF-*α* was significantly lower. In particular, expression of TNF-*α* was significantly lower for the HF/AP 5.0% group when compared to the LF group. The mRNA expression of PPAR*γ* for the HF group was significantly lower (about 90% lower) compared to the LF group. AP administration significantly accentuated this decrease in mRNA expression in a dose-dependent manner.

## 4. Discussion

The anti-obesity effects of AP, a new polyphenol, were investigated using KKAy mice as a model of obese type-II diabetes. When fed a normal diet, KKAy mice develop obesity and type-II diabetes by 12-weeks old [31], and these mice are thus widely used for research into obesity and diabetes [32, 33]. In the present study, lard was administered to 6-week-old KKAy mice for 7 weeks to induce severe obesity and diabetes and the effects of AP were evaluated. Lard is widely used in studies on obesity and diabetes [34, 35]. AP was found to significantly suppress increases in body weight and white adipose tissue weight, showing anti-obesity actions (Table 2, Figure 1). HF also increased liver fat accumulation and induced fatty liver, but AP administration lowered fat accumulation, clarifying that AP suppresses fatty liver (Table 2, Figure 2). Furthermore, measurements of plasma GOT and GPT clarified that AP suppressed fatty liver-induced hepatopathy (Table 2). Plasma glucose and insulin levels were significantly higher for the HF group than for the LF group, and severe type II diabetes was induced. AP suppressed these increases in plasma glucose and insulin levels. The HOMA-IR index, a simpler method to measure insulin sensitivity usually used in clinical and animal studies [22, 36], was significantly decreased in AP-treated groups compared to the HF group, indicating that AP reduced hyperglycemia and hyperinsulinemia (Table 2). These findings clarify that AP suppresses obesity and diabetes caused by a high-fat diet.

Obesity is caused by low energy expenditure and increased fatty acid synthesis from carbohydrates and fat intake by organs. Therefore, as well as the effects of AP on the expression of energy expenditure-related genes in skeletal muscle and liver, effects on the expression of genes related to fatty acid synthesis and fat intake in the liver were investigated.

In skeletal muscle, 7 weeks of AP administration significantly increased mRNA and protein expressions of ACO, CPT1 (*β*-oxidation enzymes) and UCP3 (uncoupling protein) compared to the HF group (Figures 3 and 4). AP administration also increased mRNA expression of PPAR*α* and PPAR*δ* (nuclear receptors). The mRNA expression of PPAR*α* increased not only in skeletal muscle, but also in liver. Activation and elevation of PPAR*α* and PPAR*δ* expression is known to increase expression of CPT1, ACO and UCP3 to elevate energy expenditure, subsequently resulting in anti-obesity actions [37–40]. This suggests that AP acts on skeletal muscle and liver to increase energy expenditure. Furthermore, AP decreased mRNA expression of ACC and FAS, the rate-limiting enzymes of fatty acid synthesis in the liver, and mRNA expression of SREBP-1c, which controls the expression of these enzymes [41]. Furthermore, AP lowered the mRNA expression of PPAR*γ* and LPL, which are related to fat intake by the liver (Figure 5). Reduced expressions of fatty acid synthetase [9], PPAR*γ* [42, 43] and LPL [44] in the liver suppress the onset of obesity and fatty liver. These findings indicate that the anti-obesity actions of AP are due to increased expression of energy expenditure-related genes in skeletal muscle and liver and decreased fatty acid synthesis and fat intake in the liver.

Insulin resistance can be generated by decreased adiponectin secretion and increased TNF-*α* secretion [45, 46]. AP increased mRNA expression of adiponectin and decreased mRNA expression of TNF-*α* in white adipose tissue (Figure 6). In white adipose tissue, AP administration significantly increased (by about 5-fold) the mRNA expression of PPAR*γ*, which facilitates the expression of adiponectin [47]. Furthermore, AP significantly increased the mRNA expression of GLUT4 in skeletal muscle (Figure 3). GLUT4 is one of the glucose transporters that are frequently expressed in skeletal muscle, and an increase in GLUT4 expression has been known to reduce insulin resistance [48]. AP thus reduces hyperglycemia and hyperinsulinemia, not simply through alleviated obesity, but through increased adiponectin secretion and suppressed TNF-*α* secretion in white adipose tissue, and increased GLUT4 expression in skeletal muscle.

**Figure 7:**
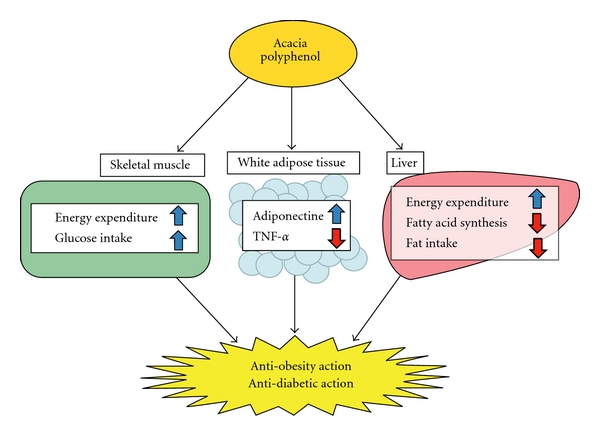
Hypothetical mechanisms of anti-obesity/diabetic actions of acacia polyphenol.

Various pharmacological actions of polyphenols have been reported. The AP used in the present study acted on skeletal muscle, liver and white adipose tissue and was shown to possess anti-obesity and anti-diabetic actions (Figure 7). While many studies have described plant extracts exhibiting anti-obesity and anti-diabetic actions, to the best of our knowledge, none have demonstrated anti-obesity and anti-diabetic actions via the modification of energy expenditure-related genes in skeletal muscle. In recent years, selective PPAR*δ* agonists, such as synthetic GW501516, have been shown to suppress body weight increase and reduce insulin resistance in obese diabetic mice [37, 38]. The present study suggests that AP is a plant extract with a novel mechanism of action with skeletal muscle as an active site.

## 5. Conclusion

In KKAy mice with high-fat diet-induced obesity, the anti-obesity actions of AP appear attributable to increased expression of energy expenditure-related genes in skeletal muscle and liver, and decreased fatty acid synthesis and fat intake in the liver. AP also reduced hyperglycemia and hyperinsulinemia by increasing adiponectin secretion and suppressing TNF-*α* secretion by white adipocytes, and elevating the expression of GLUT4 in skeletal muscle in addition to reducing obesity. In the future, AP seems to offer potential as a plant extract that is useful for alleviating metabolic syndrome.

## Figures and Tables

**Table 1:** Primer sequences of mouse mRNA.

Target	Accession number	Forward primer (5′–3′)	Reverse primer (5′–3′)	Amplicon size (bp)
PPAR*α*	NM_011144	GTACGGTGTGTATGAAGCCATCTT	GCCGTACGCGATCAGCAT	76
PPAR*δ*	NM_011145	GCCATATTCCCAGGCTGTC	CAGCACAAGGGTCATCTGTG	102
CPT1	NM_013495	GTGACTGGTGGGAGGAATAC	GAGCATCTCCATGGCGTAG	83
ACO	NM_015729	GTGCAGCTCAGAGTCTGTCCAA	TACTGCTGCGTCTGAAAATCCA	112
UCP3	NM_009464	CCAGAGCATGGTGCCTTCGCT	CTCGTGTCAGCAGCAGTG	84
GLUT4	NM_009204	GGAAGGAAAAGGGCTATGCTG	TGAGGAACCGTCCAAGAATGA	115
SREBP-1c	NM_011480	ACGGAGCCATGGATTGCACA	AAGGGTGCAGGTGTCACCTT	278
ACC	NM_133360	ATGGGCGGAATGGTCTCTTTC	TGGGGACCTTGTCTTCATCAT	148
FAS	NM_007988	TGCTCCCAGCTGCAGGC	GCCCGGTAGCTCTGGGTGTA	107
PPAR*γ*	NM_011146	CCAGAGCATGGTGCCTTCGCT	CAGCAACCATTGGGTCAGCTC	241
LPL	NM_008509	CCACAGCAGCAAGACCTTC	AGGGCGGCCACAAGTTTG	87
Adiponectin	NM_009605	GAGATGCAGGTCTTCTTGGTC	GCTCTCCTTTCCTGCCAG	105
TNF-*α*	NM_013693	AAGCCTGTAGCCCACGTCGTA	GGCACCACTAGTTGGTTGTCTTTG	122
*β*-Actin	NM_007393	GAGCGCAAGTACTCTGTGTG	CGGACTCATCGTACTCCTG	97

PPAR*α*, peroxisome proliferator-activated receptor *α*; PPAR*δ*, peroxisome proliferator-activated receptor *δ*; CPT1, carnitine palmitoyl-transferase1; ACO, acyl CoA oxidase; UCP3, uncoupling protein3; GLUT4, glucose transporter4; SREBP-1c, sterol regulatory element-binding protein-1c; ACC, acetyl-CoA carboxylase; FAS, fatty acid synthase; PPAR*γ*, peroxisome proliferator-activated receptor *γ*; LPL, lipoprotein lipase; TNF-*α*, tumor necrosis factor-*α*.

**Table 2:** Body weight gain, and plasma and hepatic biochemistry of mice fed on the experimental diets for 7 weeks.

	LF	HF	HF/AP 2.5%	HF/AP 5.0%
Food intake (g/mouse/day)	5.1 ± 0.9	4.5 ± 0.6	4.6 ± 0.5	4.4 ± 0.5
Energy intake (kcal/mouse/day)	19.4 ± 3.6	23.3 ± 3.2*	23.5 ± 2.8*	22.0 ± 2.9*
Pre-diet body weight (g)	31.3 ± 1.2	30.9 ± 1.1	31.5 ± 1.6	31.1 ± 1.0
Post-diet body weight (g)	39.7 ± 2.4	51.4 ± 2.5**	45.7 ± 4.7^∗∗,##^	39.5 ± 1.6^##^
Body weight gain (g)	8.4 ± 1.9	20.5 ± 2.0**	14.2 ± 4.3^∗∗,##^	8.4 ± 1.9^##^
Whole WAT (g)	3.14 ± 0.60	4.66 ± 0.34**	4.00 ± 0.35^∗∗,##^	3.37 ± 0.54^##^
Epididymal WAT (g)	1.63 ± 0.23	1.97 ± 0.21*	1.77 ± 0.22^#^	1.70 ± 0.22^#^
Retroperitoneal WAT (g)	0.96 ± 0.28	1.57 ± 0.21**	1.31 ± 0.19**	0.94 ± 0.19^##^
Perirenal WAT (g)	0.66 ± 0.14	1.35 ± 0.19**	0.91 ± 0.19^∗,##^	0.76 ± 0.23^##^
Liver weight (g)	1.85 ± 0.22	3.27 ± 0.44**	1.83 ± 0.40^##^	1.26 ± 0.13^∗∗,##^
Liver triglyceride (mg g^−1^ liver)	78.0 ± 13.8	156.5 ± 11.9**	82.2 ± 4.5^##^	39.5 ± 6.1^∗∗,##^
Liver cholesterol (mg g^−1^ liver)	2.3 ± 0.4	4.4 ± 0.7**	1.0 ± 0.6^∗∗,##^	0.9 ± 0.4^∗∗,##^
Glucose concentration (mmol l^−1^)	6.7 ± 2.1	20.8 ± 4.4**	8.4 ± 3.2^##^	5.6 ± 1.5^##^
Insulin concentration (*μ*U ml^−1^)	22.7 ± 16.5	171.5 ± 120.9**	42.7 ± 33.4^##^	28.8 ± 13.0^##^
HOMA-IR	7.5 ± 3.9	139.9 ± 81.6**	20.6 ± 17.3^##^	7.7 ± 5.4^##^
GOT concentration (U l^−1^)	42.7 ± 14.8	176.3 ± 77.9**	82.2 ± 59.5^#^	64.8 ± 29.6^#^
GPT concentration (U l^−1^)	16.8 ± 5.0	56.7 ± 14.0**	12.2 ± 3.0^##^	19.8 ± 7.8^##^

KKAy mice were given a low-fat diet (LF), a high-fat diet (HF), HF containing 2.5% AP (HF/AP 2.5%) or HF containing 5.0% AP (HF/AP 5.0%) for 7 weeks. After measuring body weight, white adipose tissue and liver were removed and weighed, and levels of liver triglyceride and cholesterol were measured. Blood samples were also collected to measure glucose, insulin, GOT and GPT levels. HOMA-IR was calculated from glucose and insulin levels after overnight fasting. Food intake was measured once weekly. Details of the diets are given in the “Materials and Methods” section. Data represent means ± SDs of 10 mice per group.

Tukey's test: *P  <* .*05 versus LF; **P  <* .*01 versus LF; ^#^ *P  *<* .*05 versus HF; ^##^ *P  *<* .*01 versus HF.
